# Validation of the Italian version of the 25‐item Hikikomori Questionnaire (HQ‐25‐I)

**DOI:** 10.1002/jclp.23404

**Published:** 2022-06-16

**Authors:** Emanuele Fino, Paolo Iliceto, Antonino Carcione, Eleni Giovani, Gabriella Candilera

**Affiliations:** ^1^ NTU Psychology Nottingham Trent University Nottingham UK; ^2^ S&P Statistics and Psychometrics Ltd Rome Italy; ^3^ Third Centre of Cognitive Psychotherapy Rome Italy; ^4^ Private Practice Rome Italy

**Keywords:** depression, emotional support, Hikikomori, isolation, socialization

## Abstract

**Introduction:**

The present study aimed to adapt the 25‐item Hikikomori Questionnaire to the Italian context (HQ‐25‐I) and to test its psychometric properties in two samples, particularly a sample of residents with psychiatric conditions (*n* = 117) and a sample of individuals from the community (*n* = 209).

**Methods:**

We tested the fit of the original three‐factor structure (Socialization, Isolation, and Emotional Support) and measurement invariance across the two groups, and the reliability, convergent, and criterion (concurrent) validity of the HQ‐25‐I.

**Results:**

The results showed that the original measurement model fitted the data well and that it was invariant across the two groups. The measure was reliable and positively correlated with some maladaptive personality trait domains (PID‐5‐BF), Depression (BDI‐II), and Hopelessness (BHS) in both groups, with higher scores observed in the clinical sample. However, low correlations were found between the HQ‐25‐I and the PID‐5‐BF Detachment and Negative Affectivity.

**Conclusions:**

The results from the study showed that the HQ‐25‐I is reliable, but further examination of its validity is warranted. Implications for theory and future research are discussed.

## 1 INTRODUCTION

In the last two decades, research has increasingly investigated a phenomenon seeing individuals engaging in prolonged and extreme social withdrawal and avoidance, lasting at least 6 months, raising significant public health concerns in several countries (Teo et al., 2018). In the literature, that is commonly termed as *hikikomori*, from the Japanese word indicating a condition of self‐confinement and withdrawal. Typically, hikikomori affects adolescents and young adults, with an observed average age of onset of 15 years (Pozza et al., 2019; Teo et al., 2018). Previous research showed that hikikomori is often associated with disrupted internal working model of attachment and other mental health conditions such as depression and hopelessness (Li & Wong, 2015). As a consequence, individuals affected by hikikomori tend to respond to developmental challenges, including family problems, disrupted social networking and peer interactions, and other psychosocial stressors, with extreme social and educational/occupational withdrawal (Teo et al., 2015). In particular, they have been found to typically self‐isolate in their bedrooms for months, even for years in the most extreme cases (Ferrara et al., 2020).

A recent review of the literature (Li & Wong, 2015) showed an estimated prevalence of hikikomori of about 1.2% in Japan (Koyama et al., 2010), 1.9% in Hong Kong (Wong et al., 2015), and 2.3% in Korea (Lee et al., 2018). Cases of hikikomori have been reported in Australia, Bangladesh, India, Iran, Japan, Korea, Taiwan, Thailand, and the United States (Kato et al., 2012, 2018), and recently, in European countries too (Chauliac et al., 2017; Ferrara et al., 2020; Malagón‐Amor et al., 2015). Specifically, in Italy, the prevalence of hikikomori is estimated at around 100,000 among those aged 14–25 (Poletto, 2018), about 0.8% of the population (ISTAT – Italian National Institute of Statistics, 2020). A recent study on 288 mothers and fathers from the Hikikomori Italia Parents onlus, the major national association of parents of individuals with hikikomori, reported that about the 88% of the interviewees had a male child with hikikomori, with estimated mean age of 20 years and a history of isolation spanning over 3 years (Crepaldi, 2019).

Despite hikikomori still missing a specific nomological network, to the best of our knowledge, recent research has led to significant progress in the definition and understanding of the condition and its major correlates, both from a biopsychosocial and a psychopathological perspective. In particular, a growing corpus of literature has accumulated in the last decade (Kato et al., 2018; Teo et al., 2018, 2020). The following six criteria were initially proposed by Teo and Gaw (2010) as the best to correctly identify individuals with hikikomori: (i) individuals spend most of their time, almost every day, confined to home; (ii) they markedly and persistently avoid any social situations (e.g., school and/or work) and relationships (e.g., friends and family), significantly limiting the availability of social and emotional support; (iii) their behavior affects their routine, impacting their academic and/or occupational functioning; (iv) they do not perceive their withdrawal as ego‐dystonic; (v) the condition lasts for at least 6 months; and (vi) the condition is not better explained by any alternative mental disorder (e.g., social phobia, major depression, schizophrenia, and avoidant personality disorder). However, as highlighted by Hamasaki et al. (2020), the distinction between hikikomori and other psychiatric disorders, particularly Social Anxiety Disorder (SAD) represents a key but controversial argument in the literature. In fact, previous studies reported SAD to be a common antecedent and correlate of hikikomori. Nagata et al. (2013) found that about 19% of individuals diagnosed with SAD fulfilled the aforementioned criteria for hikikomori. For this reason, Hamasaki et al. (2020) have recently argued that “it is epidemiologically clear that there is duplication but the two conditions are not identical. However, specific features unique to hikikomori are yet to be elucidated; therefore, hikikomori is not yet included in the DSM‐5.” (p. 809). Kato et al. (2020) have more recently defined hikikomori as a “form of pathological social withdrawal or social isolation whose essential feature is physical isolation in one's home” (p. 116), and the authors have proposed a refinement of the previous criteria for the identification of those with hikikomori, considering three main criteria: (i) social isolation and marked withdrawal at one's home, (ii) prolonged isolation for at least 6 months, and (iii) functional impairment and distress derived from isolation. Significant impairment has been found in the advanced phases of the condition, conversely to the earlier phases which seem to be typically characterized by a sense of contentedness and relief associated with withdrawal, with reality being perceived as painful and problematic, triggering a desire to escape.

Research on the psychopathology of hikikomori has shown that the presence of other psychiatric disorders, previously considered as exclusion criteria for a diagnosis of hikikomori (Teo & Gaw, 2010), would deserve further in‐depth investigation, and that the frequency of such comorbid conditions warrants an urgent redefinition of the nomological network of the construct. In this regard, Teo et al. (2015) identified a sample of Japanese and US college graduates with hikikomori, avoidant personality disorder, and depression as the most common psychiatric comorbid diagnoses, and those were also confirmed by other research (Frankova, 2019; Hayakawa et al., 2018), whereas Yong and Nomura (2019) found a relationship between hikikomori, hopelessness, and suicidal ideation. A recent study surveyed the attitudes of 3,262 Japanese residents aged 15–39 years and found a prevalence of hikikomori of 1.8% (Yong & Nomura, 2019). Those were more likely to self‐isolate at home, drop out of education, experience hopelessness, and attempt suicide compared to a non‐hikikomori group. Furthermore, Stip et al. (2016) have found that individuals with hikikomori might or might not experience negative symptoms in addition to social isolation, whereas prolonged sensory deprivation due to periods of withdrawal in their room could lead in some cases to a presentation of a prodromal phase of psychosis.

Research has also found a significant association between hikikomori and negative personality traits (Chong & Chan, 2012; Teo & Gaw, 2010). Recent literature (Frankova, 2019) has advanced a hypothesis about the existance of two different types of hikikomori: (i) a “primary” type, theorized as presenting no relations with personality disorders and other psychiatric comorbidities, and (ii) a “secondary” type, linked to avoidant personality, neurotic, mood, and anxiety disorders, such as major depression and social phobia, obsessive‐compulsive disorder, eating disorders, and pervasive developmental disorders. Consistently, it has been proposed that the relationships between hikikomori, trait domains, and psychopathological symptoms may vary according to the specific type of hikikomori, and in addition, as argued by Frankova (2019), the proposed differences in the psychiatric background of individuals with hikikomori may, to some extent, depend on the variability in the methodology and sampling used by those studies. However, although suggestive, such hypothesis currently lacks empirical support (Frankova, 2019).

Psychometric instruments to assess hikikomori exist. For example, the Hikikomori Behavior Checklist is a 45‐item scale validated in the Japanese context, assessing problematic behaviors by the family members of the child (Sakai et al., 2004; Uchida & Norasakkunkit, 2015). On the other hand, the NEET/Hikikomori Risk Scale (Uchida & Norasakkunkit, 2015) focused primarily on the assessment of NEET (Not in Employment Education or Training), assuming commonalities in the psychological features that can be found both in NEET and in hikikomori. However, the only instrument that to date is based on a widely accepted theoretical definition of hikikomori is the Hikikomori Questionnaire (HQ‐25; Teo et al., 2018). This is a 25‐item psychometric scale, originally validated in Japanese clinical and community samples, measuring three dimensions of Socialization, Isolation, and Emotional Support, respectively (Teo & Gaw, 2010; Teo et al., 2015). The scale was validated in two convenience samples, namely a sample of psychiatric outpatients presenting several different diagnoses and a sample of individuals from the community. Research found that the HQ‐25 has sound psychometric properties, including reliability, validity, and diagnostic accuracy (Teo et al., 2018).

It has also been hypothesized that the manifestations of hikikomori vary according to the specific context of study, due to significant cultural influences that affect the symptomatology of the condition. According to recent anecdotal reports (Hikikomori Italia, 2017), the behavioral manifestations of hikikomori in the Italian context differ to some extent to what reported in the Japanese context, ultimately showing the possible impact of cultural norms. For example, among the commonalities suggested between the Japanese and the Italian contexts, the demographic decline of the population and the increase of families with only‐children, as well as the intergenerational gap in cultural norms, religious beliefs, and adherence to traditional values, seem to play a role in determining a socio‐cultural climate of expectations and pressure that might favor the development of hikikomori. Regarding the differences between the two contexts, specifically, Italian individuals with hikikomori have been characterized by conflictual but higher frequency of interpersonal contacts with the members of their families and households, compared to their Japanese counterparts (Hikikomori Italia, 2017), the latter reported to be inclined to a comparatively higher degree of reclusion and isolation (Kato et al., 2012, 2018). For these reasons, such reports warrant further investigation and study, aiming to identify the characteristics of the manifestation of hikikomori in the Italian context, providing researchers with adequate instruments to design and perform cross‐cultural research, possibly leading to a more comprehensive overview of the phenomenon and advancement of theory, research, prevention, and intervention in the local community.

In light of the recent emergence of the phenomenon in several contexts, including European countries (Chauliac et al., 2017; Ferrara et al., 2020; Malagón‐Amor et al., 2015), and Italy, in particular (Crepaldi, 2019; Hikikomori Italia, 2017; Poletto, 2018; Ranieri et al., 2015; Sarchione et al., 2015), we believe that a psychometric assessment of hikikomori in European contexts and populations will provide researchers, clinicians, mental health practitioners, and policymakers with a useful tool to timely identify those at risk and facilitate the implementation of strategic prevention and intervention in both community and clinical settings.

The present study aimed to adapt the HQ‐25 in the Italian context (HQ‐25‐I) and to test its factor structure and reliability, construct, convergent, and concurrent (criterion) validity in two samples of Italian residents, namely (i) a sample of psychiatric patients, and (ii) a sample of individuals from the community. We hypothesized that the three‐factor measurement model found in the original HQ‐25 study in the Japanese context (Teo et al., 2018) could be adapted in the Italian context, showing invariance across the two groups, supporting the concurrent (criterion) validity of the scale. Furthermore, to test the convergent validity of the HQ‐25‐I, we used scores in five negative personality trait domains, namely Negative Affect, Detachment, Antagonism, Disinhibition, and Psychoticism, as well as with psychiatric symptoms frequently observed in hikikomori, such as Depression and Hopelessness. In the clinical sample, in light of the characteristics of the Socialization and Isolation HQ‐25 subscales, the former mainly measuring social withdrawal and lack of interest in close relationships, and the latter focusing on the tendency to isolate at home, we expected both to be highly correlated with Detachment and Negative Affectivity, two negative correlates of Extraversion (Góngora & Castro Solano, 2017). Because the Isolation subscale also contains one item assessing to what extent one tends to live by society's rules and values, we expected it to correlate moderately to highly with Antagonism, which previous studies showed to negatively correlate with Agreeableness, being characterized by a lack of motivation to maintain positive interpersonal relationships (Graziano & Eisenberg, 1997) and a dislike of others' interpersonal behavior (Lynam & Miller, 2019). Moreover, we expected that Emotional Support, mainly characterized by a perception of not being understood by others and a significant lack of social and emotional support, would highly correlate with Psychoticism, the latter including items such as “My thoughts often don't make sense to others” and “I often have thoughts that make sense to me but that other people say are strange”, and to a lesser extent, with Detachment and Negative Affectivity. Regarding Disinhibition, a trait domain expressing impulsive behavior and the experience of being seen as irresponsible, we expected lower correlations with all the HQ‐25‐I factors. Finally, in light of recent literature showing depressive symptoms in individuals with hikikomori (Hamasaki et al., 2020; Kondo et al., 2013), we hypothesized a moderate to high correlation between each of the three HQ‐25‐I subscales and Depression measured through the BDI‐II. Similarly, based on recent studies showing around 33% of individuals with hikikomori feeling hopeless compared to non‐hikikomori counterparts (about 14%; Yong & Nomura, 2019), we expected moderate correlations between the three HQ‐25‐I factors and Hopelessness measured through the BHS. In the community sample, we expected comparatively lower correlations between the HQ‐25‐I subscales and all the trait domains and symptoms.

## 2 METHODS

### 2.1 Participants and procedure

In line with the original study on the HQ‐25 (Teo et al., 2018) we recruited two convenience samples, respectively: (i) Italian residents from the community (“community” sample), and (ii) psychiatric outpatients (“clinical” sample). The community sample consisted of individuals contacted at universities, public parks, shops, markets, banks, and post offices in mid and north Italy, specifically from Lazio (mid), Abruzzo (mid), Lombardia (north), and Veneto (north). They were 209 participants, aged 18–55 (*M* = 30.2; *SD* = 9.8), of which 110 (52.6%) were women and 99 (47.4%) were men. The clinical sample included psychiatric outpatients at the Third Centre of Cognitive Psychotherapy in Rome, Italy, and other local private psychotherapy practices. They were 117 participants, aged 18–65 (*M* = 35.3; *SD* = 12.9), of which 46 (39.3%) were women and 71 (60.7%) were men. Inclusion criteria were to be resident in Italy, to be aged at least 18 years old and be fluent in Italian, with the ability to read, understand, and complete the study's procedure.

All participants were initially approached by trained staff, who explained the purpose and the characteristics of the study and invited them to take part in the study on a voluntary basis. Those who expressed their interest were asked to read, accept, and sign a written consent form to participate. Participation consisted of completing a battery of paper‐and‐pencil self‐report measures. The study was reviewed and approved by an Italian institutional ethical committee and two external reviewers, who confirmed that all procedures adhered to international standards of research involving human participants and compliance with the Code of Ethics of the World Medical Association (World Medical Association, 2013).

### 2.2 Measures

The *25‐Item Hikikomori Questionnaire* (HQ‐25; Teo et al., 2018) is a 25‐item self‐report scale measuring symptoms of hikikomori over the past 6 months. The HQ‐25 was translated from English to Italian using the English version provided in the original validation study, by two authors of the present study (Emanuele Fino and Paolo Iliceto), and the translated version was independently and blindly back‐translated by an English‐speaking translator, resulting in excellent fit to the original version. Response options for all the HQ‐25 items range from 0 (“Strongly disagree”) to 4 (“Strongly agree”). We followed the International Test Commission's (2016) guidelines for adapting tests, aiming to accomplish the following tasks: (i) evaluate any linguistic, psychological, and cultural differences in the population of interest; (ii) analyze the legitimacy of hikikomori, the construct under investigation, in the target linguistic and cultural group; and (iii) verify the suitability of the adaptation design and procedure. In particular, those were fulfilled by means of a targeted review of the literature on hikikomori in the Italian context (Crepaldi, 2019; Hikikomori Italia, 2017; Poletto, 2018; Ranieri et al., 2015; Sarchione et al., 2015), supported by independent psychological and clinical expertise, ultimately suggesting the adaptability of the final version of the scale to the Italian context.

The HQ‐25 underlies a three‐factor theoretical model of hikikomori symptoms, namely Socialization (measured by items: 1, 4, 6, 8, 11, 13, 15, 18, 20, 23, and 25), Isolation (items: 2, 5, 9, 12, 16, 19, 22, and 24), and Emotional support (items: 3, 7, 10, 14, 17, and 21). A total HQ‐25 score was obtained by summing up individual items' scores. Research showed that the HQ‐25 is internally consistent, with Cronbach's *α* values of 0.96, 0.94, 0.91, and 0.88, for the total scale and the three subscales, respectively (Teo et al., 2018). The HQ‐25‐I is provided in Appendix A.

The *Personality Inventory for DSM‐5 – Brief Form* (PID‐5‐BF; American Psychiatric Association, 2013), Italian version (Fossati et al., 2013), is a shortened version of the original 220‐item *Personality Inventory for DSM‐5* (PID; American Psychiatric Association, 2013). The PID‐5‐BF includes 25 items rated from 0 ("Very false or often false") to 3 ("Very true or often true"), measuring five maladaptive personality trait domains, namely Negative Affectivity, Detachment, Antagonism, Disinhibition, and Psychoticism. Each domain is measured by five items, for a total of five domain scales. Research in the Italian context showed Cronbach's alpha values exceeding 0.90 for all the domain scales (Fossati et al., 2013). In the present sample, all the subscales of the PID‐5‐BF were internally consistent, showing the following values of Cronbach's *α*: 0.91 (Negative Affectivity), 0.83 (Detachment), 0.86 (Antagonism), 0.83 (Disinhibition), and 0.81 (Psychoticism).

The *Beck Depression Inventory‐II* (BDI‐II; Beck et al., 1996), Italian version (Ghisi et al., 2006), is a 21‐item self‐report inventory assessing the severity of depressive symptomatology, asking respondents to select statements that reflect how they have felt over the last 2 weeks. BDI‐II scores range between 0 and 63, with higher scores indicating more severe symptomatology. Previous findings on the Italian version of the scale indicated that the scale is internally consistent, with Cronbach's *α* values ranging from 0.80 to 0.85, and that the scale showed convergent, divergent, and criterion validity (Ghisi et al., 2006; Iliceto et al., 2016). In the present sample, the BDI‐II showed a satisfactory value of Cronbach's *α* (0.81), indicating internal consistency.

The *Beck Hopelessness Scale* (BHS; Beck & Steer, 1988), Italian version (Iliceto et al., 2009), is a 20‐item self‐report measure of hopelessness, a construct frequently described in the literature in terms of a major predictor and a proxy of suicidal ideation (Beck & Steer, 1988; Iliceto, D'Antuono, et al., 2020). The current study used the Likert‐type 5‐point response format described in recent literature (Iliceto & Fino, 2015), with all items scored from 0 ("Strongly disagree") to 4 ("Strongly agree"). BHS total scores range from 0 to 80, with higher scores indicating higher hopelessness and risk for suicidal ideation. Recent studies using the Italian version of the scale showed that the scale is internally consistent, with Cronbach's *α* values ranging from 0.81 to 0.89 (Fino et al., 2014; Iliceto & Fino, 2015; Iliceto et al., 2014; Iliceto, Fino, et al., 2020). In the present sample, the BHS showed a satisfactory value of Cronbach's *α* (0.84), suggesting that the scale was internally consistent.

### 2.3 Statistical analyses

We used confirmatory factor analysis (CFA) to test the factor structure of the HQ‐25‐I. CFA tests the latent structure of a set of observed variables by fitting a theoretical model to the observed data and evaluating the goodness of the relevant fit. In the present study, we specified and evaluated the fit of the theoretical latent model originally developed in the Japanese context for the HQ‐25 (Teo et al., 2018), and we used the empirical variance‐covariance matrix to run CFA with maximum likelihood estimation (Bollen, 1989; Brown, 2015; Byrne et al., 1989). We tested the distributional assumptions of univariate normality by evaluating indices of skewness and kurtosis across all the observed variables and of multivariate normality by estimating Mardia's coefficient, using *v* (*v* + 2) as a cutoff value (where *v* represents the number of observed variables; see Bollen, 1989), and Mahalanobis distances, specifically the distribution of distances between observed values and the centroid of the means computed across all the variables in the model, considering values with *p* < 0.001 as possible multivariate outliers (Tabachnick & Fidell, 2007). We evaluated the fit of the model by using the following fit indices (and cutoff values; Brown, 2015; Kenny, 2015): the Comparative Fit Index (CFI > 0.95), the Tucker–Lewis Index (TLI > 0.95), the Root Mean Square Error of Approximation (RMSEA < 0.06), and the Standardized Root Mean Square Residual (SRMR < 0.08). To test the reliability of latent factors we used McDonald's omega, considering values greater than 0.75 as indicative of satisfactory reliability (McDonald, 1999; McNeish, 2018).

We used multigroup CFA to test for the measurement invariance of the HQ‐25‐I across the community and the clinical sample (Vandenberg & Lance, 2000). The method requires to run a series of nested CFA models and to progressively constrain parameters to be equal across groups, aiming to compare the fit of each nested model to its less‐constrained antecedent (Byrne, 2010). First, we tested a model with unconstrained parameters in both samples (configural model). Second, we tested a model with factor loadings constrained to be equal across both samples, and we compared its fit to the fit obtained on the configural model (metric invariance). Third, we constrained observed intercepts to be equal across the groups, whereas latent means were fixed to zero in one group but were freely estimated in the other group, and we compared the resulting model's fit to the fit of the metric invariant model (scalar invariance). Fourth, we additionally constrained residual variances and we compared the fit of the resulting model to the fit of the scalar invariant model (strict invariance). We used the change in CFI (ΔCFI ≤ −0.005), RMSEA (ΔRMSEA ≥ 0.10), and SRMR (ΔSRMR ≥ 0.025 for metric; ≥0.005 for scalar and strict) across the models to evaluate whether the HQ‐25‐I was invariant, at each of the aforementioned stages of the multigroup analysis (e.g., see Chen, 2007; Floyd & Widaman, 1995; Little, 1997; Meredith, 1993). We used Pearson's product‐moment correlation coefficient for convergent validity analyses. CFA and multigroup analyses were conducted in R (R Core Team, 2021) and lavaan (Rosseel, 2012). All the other analyses were conducted in JASP 0.14.1 (JASP Team, 2020).

## 3 RESULTS

In the community sample, we found no statistically significant age differences ($t_{\left(207\right)}$ = 1.78; *p* = 0.07) between women (*M* = 29.1; *SD* = 10.2) and men (*M* = 31.5; *SD* = 9.2), and no gender differences across groups identified by working status ($\chi_{\left(1\right)}^{2}$ = 1.3; *p* = 0.24) or civil status ($\chi_{\left(1\right)}^{2}$ = .45; *p* = 0.50). Men (*n* = 52) presenting fewer than 18 years of education were less numerous than women (*n* = 34), with the difference being statistically significant ($\chi_{\left(2\right)}^{2}$ = 10.1, *p* < 0.007). Table 1 reports detailed socio‐demographic characteristics for the community sample. No socio‐demographic characteristics were obtained for the psychiatric sample, which was composed of individuals presenting the following main diagnoses: Antisocial personality disorder (1, 0.9%), avoidant personality disorder (1, 0.9%), histrionic personality disorder (2, 1.7%), obsessive‐compulsive personality disorder (3, 2.6%), other unspecified disorder (4, 3.4%), paranoid personality disorder (4, 3.4%), gambling disorder (5, 4.3%), generalized anxiety disorder (5, 4.3%), major depressive disorder (5, 4.3%), narcissistic personality disorder (6, 5.1%), unspecified personality disorder (7, 6%), unspecified mood disorder (8, 6.8%), borderline personality disorder (11, 9.4%), alcohol use disorder (15, 12.8%), social phobia (16, 13.7%), and passive‐aggressive personality disorder (24, 20.5%),

**Table 1:** Community sample, demographic characteristics of participants (*n* = 209)

	Men (*n* = 99)	Women (*n* = 110)	Statistics	*p*
**Age** a				
Mean (*SD*)	31.5 (9.2)	29.1 (10.2)	$t_{\left(207\right)}\;$= 1.79	0.07
**Working status** b				
University students	22 (22.2)	32 (29.1)	$\chi_{\left(7\right)}^{2}\;$= 23.8	0.001
Unemployed	33 (33.3)	39 (35.5)		
Industry workers	4 (4.0)	13 (11.8)		
Employees	33 (33.3)	39 (35.5)		
Retailers	4 (4.0)	10 (9.1)		
Professionals	9 (9.1)	2 (1.8)		
Entrepreneurs	22 (22.2)	6 (5.5)		
Teachers	1 (1.0)	3 (2.7)		
**Education** b				
≤8 years	3 (3.0)	4 (3.6)	$\chi_{\left(2\right)}^{2}$ = 10.1	0.006
≤13 years	44 (44.4)	72 (65.5)		
≤18 years	52 (52.5)	34 (30.9)		
**Marital status** b				
Unmarried	76 (76.8)	80 (72.7)	$\chi_{\left(1\right)}^{2}\;$= 0.45	0.50
Married	23 (23.2)	30 (27.3)		

^a^ Values expressed as mean (*SD*).

^b^ Values expressed as *n* (%).

We estimated skewness and kurtosis for all the HQ‐25‐I items, and we found that all the values were comprised between −1 and +1, indicating that the data approximated the univariate normal distribution. Before running CFA, we tested for the multivariate distributional assumptions of the model by means of Mardia's coefficient. The results showed that the coefficient was equal to 523.22, a value lower than the cutoff (675), indicating that the data approximated the multivariate normal distribution. Moreover, using the *χ* ^*2*^ distribution, we estimated Mahalanobis' distances and we calculated the relevant critical values. The results showed no multivariate outliers. Based on such evidence, we proceeded with the CFA.

We tested the HQ‐25 three‐factor measurement model by means of CFA, with each item loading onto its relevant factor as per the original validation article of the questionnaire in the Japanese context (Teo et al., 2018). The model achieved satisfactory fit in both the community sample (CFI = 0.98, TLI = 0.97, RMSEA = 0.04, SRMR = 0.03) and the clinical sample (CFI = 1.00, TLI = 0.99, RMSEA = 0.02, SRMR = 0.04). In both samples, items' standardized regression weights ranged from 0.76 to 0.85, whereas their squared multiple correlations, indicating the amount of variance in the observed variables that is accounted for by the latent factors, ranged from 0.57 to 0.72 (Table 2).

**Table 2:** Confirmatory factor analysis, standardized regression weights, and squared multiple correlations

Subscales	HQ‐25‐I items	Community sample (*n* = 209)		Clinical sample (*n* = 117)	
		Standardized regression weights	Squared multiple correlations	Standardized regression weights	Squared multiple correlations
Socialization	Item 1	0.812	0.659	0.755	0.570
	Item 4	0.824	0.679	0.794	0.630
	Item 6	0.803	0.645	0.799	0.638
	Item 8	0.822	0.676	0.847	0.718
	Item 11	0.834	0.696	0.766	0.587
	Item 13	0.822	0.676	0.781	0.610
	Item 15	0.813	0.660	0.795	0.632
	Item 18	0.822	0.676	0.802	0.644
	Item 20	0.820	0.672	0.788	0.622
	Item 23	0.827	0.684	0.811	0.657
	Item 25	0.831	0.691	0.839	0.704
Isolation	Item 2	0.783	0.613	0.797	0.636
	Item 5	0.799	0.639	0.779	0.606
	Item 9	0.790	0.625	0.769	0.591
	Item 12	0.791	0.626	0.763	0.582
	Item 16	0.790	0.623	0.789	0.623
	Item 19	0.801	0.642	0.776	0.602
	Item 22	0.779	0.607	0.771	0.595
	Item 24	0.796	0.634	0.793	0.629
Emotional Support	Item 3	0.795	0.632	0.760	0.577
	Item 7	0.810	0.657	0.816	0.666
	Item 10	0.816	0.666	0.762	0.580
	Item 14	0.823	0.678	0.773	0.598
	Item 17	0.767	0.589	0.794	0.631
	Item 21	0.784	0.614	0.783	0.613

Omega values indicated satisfactory reliability for the HQ‐25‐I subscales (in both the community and the clinical sample, respectively): Socialization (0.83 and 0.86), Isolation (0.82 and 0.87), and Emotional Support (0.78 and 0.81). When testing for invariance, we found satisfactory fit across all the nested models, with negligible changes in fit indices, in both samples. Table 3 reports detailed results from the CFA and the fit indices obtained across all the models and samples.

**Table 3:** Tests of measurement invariance (multigroup CFA), goodness‐of‐fit indices

	df	CFI	TLI	RMSEA	RMSEA 95% CI		SRMR	Δdf	ΔCFI	ΔTLI	ΔRMSEA	ΔSRMR
Models					Lower	Upper						
Baseline community sample	272	0.976	0.973	0.041	0.029	0.051	0.029					
Baseline clinical sample	272	0.995	0.994	0.018	0	0.041	0.035					
Configural	544	0.982	0.98	0.034	0.023	0.044	0.031					
Metric	566	0.985	0.984	0.031	0.017	0.041	0.032	22	0.003	0.004	−0.004	0.001
Scalar	588	0.989	0.989	0.026	0.008	0.037	0.032	22	0.004	0.004	−0.005	0
Strict	613	0.991	0.991	0.023	0	0.034	0.032	25	0.002	0.003	−0.003	0

We found moderate subscale inter‐correlations, respectively in the community sample and the clinical sample, between Socialization and Isolation (*r* = 0.38, *p* < 0.001; *r* = 0.40, *p* < 0.001), Socialization and Emotional Support (*r* = 0.39, *p* < 0.001; *r* = 0.41, *p* < 0.001), and between Isolation and Emotional Support (*r* = 0.38, *p* < 0.001; *r* = 0.40, *p* < 0.001).

Regarding convergent validity, we found moderate correlations between HQ‐25‐I subscale scores and personality negative trait domains (PID‐5‐BF), Depression (BDI‐II), and Hopelessness (BHS) in the clinical sample, with all correlations being statistically significant. Particularly, in the clinical sample, we found positive and moderate‐to‐high correlations between total‐HQ‐25‐I scores and Socialization, Isolation, and Emotional support, respectively with Antagonism, Disinhibition, Psychoticism, Depression, and Hopelessness. Positive but lower correlations were found with Negative Affectivity and Detachment. As hypothesized, in the community sample, the correlations between the HQ‐25‐I total and subscale scores and the external variables were positive and low to null (and mostly nonsignificant). All the correlations are reported in Table 4.

**Table 4:** HQ‐25‐I convergent validity analyses

Community sample (*n* = 209)	HQ‐25 total score	Socialization	Isolation	Emotional support
Negative affectivity (PID‐5‐BF)	0.203**	0.183**	0.175*	0.098
Detachment (PID‐5‐BF)	0.009	0.036	0.03	0.005
Antagonism (PID‐5‐BF)	0.175*	0.176*	0.104	0.128
Disinhibition (PID‐5‐BF)	0.035	0.071	0.038	0.036
Psychoticism (PID‐5‐BF)	0.067	0.118	0.015	0.004
Hopelessness (BHS)	0.006	0.001	0.034	0.05
Depression (BDI‐II)	0.061	0.09	0.035	0.052

**Clinical sample (** ***n*** = **117)**	**HQ‐25 total score**	**Socialization**	**Isolation**	**Emotional support**
Negative affectivity (PID‐5‐BF)	0.251**	0.198*	0.293**	0.179
Detachment (PID‐5‐BF)	0.096	0.088	0.052	0.16
Antagonism (PID‐5‐BF)	0.507**	0.454**	0.510**	0.367**
Disinhibition (PID‐5‐BF)	0.358**	0.384**	0.252**	0.294**
Psychoticism (PID‐5‐BF)	0.500**	0.486**	0.462**	0.326**
Hopelessness (BHS)	0.387**	0.339**	0.412**	0.254**
Depression (BDI‐II)	0.462**	0.450**	0.335**	0.498**

*Note*: All values expressed in terms of Pearson's product‐moment correlation coefficient (*r*).

* *p* < 0.01

** *p* < 0.001.

## 4 DISCUSSION

The present study adapted the HQ‐25 to the Italian context (HQ‐25‐I) and tested its factor structure and construct validity, reliability, convergent and criterion (concurrent) validity, in two samples of Italian residents, namely (i) a sample of psychiatric patients, and (ii) a sample of individuals from the community. Based on a commonly accepted and widespread definition of hikikomori (Teo & Gaw, 2010) and recent research findings (Chong & Chan, 2012; Yong & Nomura, 2019), we hypothesized that the original three‐factor model developed and validated in the Japanese context (Teo et al., 2018) could be adapted to the Italian context in both groups, showing measurement invariance across them. Furthermore, in the clinical sample, we expected Socialization and Isolation to highly correlate with Detachment, Negative Affectivity, and Antagonism, and Emotional Support with Psychoticism, Detachment, and Negative Affectivity, respectively. Regarding Disinhibition, we expected positive but lower correlations with all the HQ‐25‐I factors. We also hypothesized moderate to high correlations between each of the three HQ‐25‐I subscales and Depression measured through the BDI‐II and Hopelessness measured through the BHS. In the community sample, we expected comparatively lower correlations between the HQ‐25 subscales and all the trait and symptom domains.

Our findings showed that the original measurement model of the HQ‐25 (Teo et al., 2018) was a good fit to the data, with all factor loadings being high and the three original subscales being reliable. Metric, scalar, and strict invariance tested across the clinical sample and the community sample were established, providing evidence of the validity of the HQ‐25‐I in both clinical and nonclinical samples. However, our hypotheses on the correlations between HQ‐25‐I scores, trait domains, and symptoms were only partially confirmed. In particular, the low correlations between each of the HQ‐25‐I factors, Detachment, and Negative Affectivity were unexpected and challenged our initial interpretation. It must be noted that the PID‐5‐BF measures Detachment in terms of individuals' tendency to feel like nothing they do really matters and to steer clear of romantic relationships, no interest in friends nor in getting too close to people, and rarely getting enthusiastic about anything (American Psychiatric Association, 2013), tapping into pathological low extraversion. Furthermore, the HQ‐25‐I factors were all moderately to highly correlated with Antagonism, Psychoticism, Depression, and Hopelessness, and to a lesser extent with Dishinibition. Given such results, we recommend future research to focus on improving the understanding of the cognitive, emotional, and psychopathological correlates of hikikomori, in particular those that distinguish the condition from other syndromes, and in the same vein, to clarify whether the hypothesis of primary versus secondary hikikomori is empirically justified, and under which circumstances (Frankova, 2019). Such evidence would help improve our understanding of the characteristics of hikikomori and shed a light on the validity of the HQ‐25, with great benefit for theory and clinical psychological research.

However, the low correlations between Detachment and each of the HQ‐25‐I factors, and Socialization, in particular, might indicate that this component of hikikomori was not sufficiently present in this sample, with the recruitment strategy possibly not having reached individuals with a sufficient degree of social withdrawal and isolation, although we cannot exclude problems with the cross‐cultural and conceptual translation of hikikomori into the Italian context, either. In any case, the evidence here presented challenges the construct validity of the instrument in a key area of hikikomori, such that it does not appear to measure the breadth of the condition as it has been defined previously in the literature. This necessarily has implications for any use of this translation. For this reason, we believe that further research on the construct validity of the HQ‐25 will need to clarify this important aspect, which is of foremost importance for future applications of the instrument. In fact, if confirmed, the adaptation of the HQ‐25 in the Italian context would allow researchers to carry out cross‐cultural studies on hikikomori, possibly enabling them to better investigate the withdrawal and isolation experienced by those individuals and if and how those vary across cultural contexts and settings, and to shed a light on the condition and its possible associations with personality trait domains and depressive symptoms. Regarding the latter aspect, we consider the observed correlations between hikikomori, Depression, and Hopelessness also of great interest, as they confirm results from previous research (Hamasaki et al., 2020; Kondo et al., 2013), and require further investigation of the role of hopelessness as a potential predictor of suicidal ideation in hikikomori (Yong & Nomura, 2019). In this regard, a recent study (Tateno et al., 2019) has hypothesized that depression might represent a “gateway condition” for hikikomori (p. 8). We believe that the analysis of hopeless depression as a possible predictor of hikikomori has great theoretical and clinical potential, and we think that these constructs should be analyzed in relation to dysfunctional attachment and environmental triggers that might lead some individuals to social withdrawal, exploring the latter as a potentially failed solution to the challenge to bond, and use such evidence to better define a developmental model of hikikomori (Krieg & Dickie, 2013).

Moreover, trait domains and symptoms could be targeted in psychotherapy and other established psychiatric interventions, thus providing individuals with hikikomori with efficacious assessment and treatment. However, as already discussed for personality, although we believe this could represent a promising future research area to gain a deeper understanding of the psychopathology of hikikomori as measured through the HQ‐25, further evidence is needed to support the construct, convergent, and discriminant validity of the HQ‐25‐I, and we recommend future research to shed a light on this important question.

## 5 CONCLUSIONS

The results from the current study fully confirmed our hypotheses on the reliability of the HQ‐25, but not on its validity. We believe these findings carry important advantages and implications: First, they confirm the reliability of a single measure of hikikomori in the Italian context, potentially contributing to improve our understanding of important emerging mental health conditions, globally, and providing practitioners and researchers from diverse contexts with preliminary evidence on the psychometric properties of the instrument in the Italian context. Second, they provide evidence on hikikomori a complex psychological phenomenon, requiring future research endeavors and possibly cross‐cultural studies to clarify its dynamics and relations with dimensions of personality, attachment, and psychiatric comorbidities, and to confirm the construct validity of the HQ‐25 in the Italian context. Particularly, we recommend future research to use representative sampling, targeting those with high scores in SAD, Detachment, and avoidant personality, to specifically address the relationship between hikikomori and those trait domains and conditions. In addition, future studies should clarify the specific relationships between hikikomori, Detachment, and Negative Affectivity, which in the current study were found to be lowly correlated, in both community and clinical samples.

This study has limitations. First, as already mentioned, it used two convenience and nonrepresentative samples (e.g., the community sample was recruited from a variety of public places, and yet hikikomori describes individuals who stay at home and avoid others, leading to possible sample selection bias), and in addition, the sample size was limited, particularly for the clinical sample, requiring future research to reproduce the analyses in larger representative clinical and community samples. Second, it was based on self‐reports, limiting the validity of the results. Third, it did not test for the validity of the HQ‐25‐I in relation to measures of social isolation and withdrawal, and this is an important point for which future research is warranted. Fourth, it did not test for the diagnostic accuracy of the HQ‐25‐I, and such aspect requires further investigation for a proper application of the questionnaire in clinical settings in the Italian context. Finally, we think that future research would benefit from investigating the relations between the HQ‐25‐I and measures of lack of interest in close relationships (Teo et al., 2018), social, educational, and occupational engagement (Teo et al., 2015), and sensation seeking (Loscalzo et al., 2020), all considered in the literature as divergent behaviors in relation to hikikomori.

## CONFLICT OF INTEREST

The authors declare no conflicts of interest.

## ETHICS STATEMENT

All the procedures followed in the study adhered to international standards of research involving human participants and compliance with the Code of Ethics of the World Medical Association (World Medical Association, 2013). All participants gave their informed consent before their inclusion in the study.

## Data Availability

The data that support the findings of this study are available from the corresponding author upon reasonable request.

## ITALIAN VERSION OF THE 25‐ITEM HIKIKOMORI QUESTIONNAIRE HQ‐25 (HQ‐25‐I)

### 1

**Table:**

Over the last 6 months, how accurately do the following statements describe you?						
*(Quanto ritiene che le seguenti affermazioni la descrivano, con riferimento agli ultimi sei mesi?)*						
Items		Strongly disagree *(Fortemente in disaccordo)*	Somewhat disagree *(Abbastanza in disaccordo)*	Neither agree nor disagree *(Né d'accordo né in disaccordo)*	Somewhat Agree *(Abbastanza d'accordo)*	Strongly Agree *(Fortemente d'accordo)*
1	I stay away from other people. *(Sto lontano dalle altre persone.)*	0	1	2	3	4
2	I spend most of my time at home. *(Passo la maggior parte del mio tempo a casa.)*	0	1	2	3	4
3	There really isn't anyone with whom I can discuss matters of importance. *(Non c'è davvero nessuno con cui posso discutere di questioni importanti.)*	0	1	2	3	4
4 (R)	I love meeting new people. *(Adoro incontrare nuove persone.)*	0	1	2	3	4
5	I shut myself in my room. *(Mi chiudo nella mia stanza.)*	0	1	2	3	4
6	People bother me. *(Mi infastidiscono le persone.)*	0	1	2	3	4
7 (R)	There are people in my life who try to understand me. *(Ci sono persone nella mia vita che cercano di capirmi.)*	0	1	2	3	4
8	I feel uncomfortable around other people. *(Mi sento a disagio attorno alle altre persone.)*	0	1	2	3	4
9	I spend most of my time alone. *(Passo la maggior parte del mio tempo da solo.)*	0	1	2	3	4
10 (R)	I can share my personal thoughts with several people. *(Posso condividere i miei pensieri personali con diverse persone.)*	0	1	2	3	4
11	I don't like to be seen by others. *(Non mi piace essere visto dagli altri.)*	0	1	2	3	4
12	I rarely meet people in‐person. *(Raramente incontro gente di persona.)*	0	1	2	3	4
13	It is hard for me to join in on groups. *(È difficile per me unirmi a dei gruppi.)*	0	1	2	3	4
14	There are few people I can discuss important issues with. *(Ci sono poche persone con cui posso discutere di questioni importanti.)*	0	1	2	3	4
15 (R)	I enjoy being in social situations. *(Mi piace stare in situazioni sociali.)*	0	1	2	3	4
16	I do not live by society's rules and values. *(Non vivo secondo le regole e i valori della società.)*	0	1	2	3	4
17	There really isn't anyone very significant in my life. *(Non c'è davvero nessuno di molto importante nella mia vita.)*	0	1	2	3	4
18	I avoid talking with other people. *(Evito di parlare con altre persone.)*	0	1	2	3	4
19	I have little contact with other people talking, writing, and so on. *(Ho pochi contatti con altre persone, sia che si tratti di parlare, scrivere, e così via.)*	0	1	2	3	4
20	I much prefer to be alone than with others. *(Preferisco di gran lunga stare da solo che con gli altri.)*	0	1	2	3	4
21 (R)	I have someone I can trust with my problems. *(Ho qualcuno di cui posso fidarmi per quanto riguarda i miei problemi.)*	0	1	2	3	4
22 (R)	I rarely spend time alone. *(Raramente passo del tempo da solo.)*	0	1	2	3	4
23	I don't enjoy social interactions. *(Non mi piacciono le interazioni sociali.)*	0	1	2	3	4
24	I spend very little time interacting with other people. *(Passo pochissimo tempo a interagire con altre persone.)*	0	1	2	3	4
25 (R)	I strongly prefer to be around other people. *(Preferisco decisamente stare in mezzo alle altre persone.)*	0	1	2	3	4

*Note*: The HQ‐25 has a theoretical score range of 0–100.

Socialization: items 1, 4, 6, 8, 11, 13, 15, 18, 20, 23, and 25.

Isolation: items 2, 5, 9, 12, 16, 19, 22, and 24.

Emotional Support: items 3, 7, 10, 14, 17, and 21.

*(L'HQ‐25 ha un range di punteggi teorici che varia da 0 a 100*.

*Socializzazione: items 1, 4, 6, 8, 11, 13, 15, 18, 20, 23, and 25*.

*Isolametno: item 2, 5, 9, 12, 16, 19, 22, and 24*.

*Supporto Emotivo: item 3, 7, 10, 14, 17, and 21.)*

(R) The item requires reverse‐scoring.

*((R) Item i cui punteggi vanno invertiti.)*
